# PTPN22 Association in Systemic Lupus Erythematosus (SLE) with Respect to Individual Ancestry and Clinical Sub-Phenotypes

**DOI:** 10.1371/journal.pone.0069404

**Published:** 2013-08-07

**Authors:** Bahram Namjou, Xana Kim-Howard, Celi Sun, Adam Adler, Sharon A. Chung, Kenneth M. Kaufman, Jennifer A. Kelly, Stuart B. Glenn, Joel M. Guthridge, Robert H. Scofield, Robert P. Kimberly, Elizabeth E. Brown, Graciela S. Alarcón, Jeffrey C. Edberg, Jae-Hoon Kim, Jiyoung Choi, Rosalind Ramsey-Goldman, Michelle A. Petri, John D. Reveille, Luis M. Vilá, Susan A. Boackle, Barry I. Freedman, Betty P. Tsao, Carl D. Langefeld, Timothy J. Vyse, Chaim O. Jacob, Bernardo Pons-Estel, on behalf of the Argentine Collaborative Group, Timothy B. Niewold, Kathy L. Moser Sivils, Joan T. Merrill, Juan-Manuel Anaya, Gary S. Gilkeson, Patrick M. Gaffney, Sang-Cheol Bae, Marta E. Alarcón-Riquelme, on behalf of the BIOLUPUS and GENLES networks, John B. Harley, Lindsey A. Criswell, Judith A. James, Swapan K. Nath

**Affiliations:** 1 Arthritis and Clinical Immunology Research Program, Oklahoma Medical Research Foundation, Oklahoma City, Oklahoma, United States of America; 2 Cincinnati Children's Hospital Medical Center, Cincinnati, Ohio, United States of America; 3 Rosalind Russell Medical Research Center for Arthritis, Department of Medicine, University of California San Francisco, San Francisco, California, United States of America; 4 US Department of Veterans Affairs Medical Center, Cincinnati, Ohio, United States of America; 5 Department of Medicine, University of Oklahoma Health Sciences Center, Oklahoma City, Oklahoma, United States of America; 6 Department of Medicine, University of Alabama at Birmingham, Birmingham, Alabama, United States of America; 7 Department of Rheumatology, Hanyang University Hospital for Rheumatic Diseases, Seoul, Korea; 8 Division of Rheumatology, Northwestern University Feinberg School of Medicine, Chicago, Illinois, United States of America; 9 Department of Medicine, Johns Hopkins University School of Medicine, Baltimore, Maryland, United States of America; 10 Department of Internal Medicine, University of Texas Health Science Center at Houston, Houston, Texas, United States of America; 11 Department of Medicine, University of Puerto Rico Medical Sciences Campus, San Juan, Puerto Rico; 12 Division of Rheumatology, University of Colorado School of Medicine, Aurora, Colorado, United States of America; 13 Center for Public Health Genomics and Department of Biostatistical Sciences, Wake Forest University Health Sciences, Wake Forest, North Carolina, United States of America; 14 Division of Rheumatology, University of California Los Angeles, Los Angeles, California, United States of America; 15 Department of Biostatistical Sciences, Wake Forest University Health Sciences, Wake Forest, North Carolina, United States of America; 16 Divisions of Genetics and Molecular Medicine and Immunology, King's College London, London, United Kingdom; 17 Department of Medicine, Keck School of Medicine, University of Southern California, Los Angeles, California, United States of America; 18 Department of Medicine, Sanatorio Parque, Rosario, Argentina; 19 Division of Rheumatology and Department of Immunology, Mayo Clinic, Rochester, Minnesota, United States of America; 20 Clinical Pharmacology, Oklahoma Medical Research Foundation, Oklahoma City, Oklahoma, United States of America; 21 Center for Autoimmune Diseases Research, Universidad del Rosario, Bogota, Colombia; 22 Department of Medicine, Division of Rheumatology, Medical University of South Carolina, Charleston, South Carolina, United States of America; 23 Centro de Genómica e Investigación Oncológica (GENYO) Pfizer-Universidad de Granada-Junta de Andalucía, Granada, Spain; University of Patras Medical School, Greece

## Abstract

Protein tyrosine phosphatase non-receptor type 22 (*PTPN22*) is a negative regulator of T-cell activation associated with several autoimmune diseases, including systemic lupus erythematosus (SLE). Missense rs2476601 is associated with SLE in individuals with European ancestry. Since the rs2476601 risk allele frequency differs dramatically across ethnicities, we assessed robustness of *PTPN22* association with SLE and its clinical sub-phenotypes across four ethnically diverse populations. Ten SNPs were genotyped in 8220 SLE cases and 7369 controls from in European-Americans (EA), African-Americans (AA), Asians (AS), and Hispanics (HS). We performed imputation-based association followed by conditional analysis to identify independent associations. Significantly associated SNPs were tested for association with SLE clinical sub-phenotypes, including autoantibody profiles. Multiple testing was accounted for by using false discovery rate. We successfully imputed and tested allelic association for 107 SNPs within the *PTPN22* region and detected evidence of ethnic-specific associations from EA and HS. In EA, the strongest association was at rs2476601 (P = 4.7×10^−9^, OR = 1.40 (95% CI = 1.25–1.56)). Independent association with rs1217414 was also observed in EA, and both SNPs are correlated with increased European ancestry. For HS imputed intronic SNP, rs3765598, predicted to be a *cis*-eQTL, was associated (P = 0.007, OR = 0.79 and 95% CI = 0.67–0.94). No significant associations were observed in AA or AS. Case-only analysis using lupus-related clinical criteria revealed differences between EA SLE patients positive for moderate to high titers of IgG anti-cardiolipin (aCL IgG >20) versus negative aCL IgG at rs2476601 (P = 0.012, OR = 1.65). Association was reinforced when these cases were compared to controls (P = 2.7×10^−5^, OR = 2.11). Our results validate that rs2476601 is the most significantly associated SNP in individuals with European ancestry. Additionally, rs1217414 and rs3765598 may be associated with SLE. Further studies are required to confirm the involvement of rs2476601 with aCL IgG.

## Introduction

Protein tyrosine phosphatase non-receptor type 22 (*PTPN22*) is a powerful negative regulator of T-cell activation. This gene is strongly associated with systemic lupus erythematosus (SLE) [1], [2], [3] as well as other autoimmune diseases including type 1 diabetes [4], rheumatoid arthritis [5], juvenile idiopathic arthritis [6], autoimmune thyroid disease [7], granulomatosis with polyangiitis (formerly known as Wegener's granulomatosis) [8], myasthenia Gravis [9], Hashimotos thyroiditis [10], Graves' disease [11], Addison's disease [12], systemic sclerosis [13], and generalized vitiligo [14]. *PTPN22* has strong functional consequences for immune responses, supporting evidence of association with SLE and other autoimmune diseases [4], [5], [6], [7], [8], [9], [10], [11], [12], [13], [14], [15], [16], [17], [18], [19], [20], [21], [22], [23], [24], [25], [26], [27].

*PTPN22* is located on chromosome 1 and codes for the lymphoid specific tyrosine phosphatase protein, Lyp. This protein is comprised of 807 amino acids, and contains an N-terminal protein tyrosine phosphatase (PTP) domain and four proline-rich motifs (P1–4) in the C-terminal region. Lyp has a major role in regulation of the Src family of tyrosine kinases (SFKs). SFKs serve as molecular switches that regulate a variety of cellular events including cell growth, division, differentiation and programmed death. The P1 proline-rich motif of Lyp is important for its interaction with the C-terminal Src tyrosine kinase (CSK), a negative regulatory kinase [28]. Csk phosphorylates C-terminal tyrosines in SFKs, and the Lyp-CSK interaction destabilizes the kinase domains of SFKs through tyrosine dephosphorylation. The well established *PTPN22* polymorphism (rs2476601) encodes the amino acid substitution of arginine to tryptophan at position 620 (R620W) which disrupts the Lyp-Csk interaction and therefore has been the main focus of many research studies [26].

SLE prevalence varies substantially by ethnic ancestry: African-Americans have a 3 to 5 fold increased risk of SLE compared to individuals with European ancestry [29], [30], [31]. Earlier studies report association between *PTPN22* and SLE, primarily with the non-synonymous variant rs2476601 in European-derived populations. However, the frequency of this disease-associated SNPs varies strongly among Europeans based on geographic region, with a higher prevalence in Northern European countries relative to Southern European populations [32], [33]. Additionally, rs2476601 is either monomorphic or extremely rare in the Japanese population and only rarely observed among the admixed African-American population [32].

In this study we investigated association *PTPN22* variants in SLE cases and healthy controls in four diverse ethnic populations in order to elucidate the population specific relationship between different *PTPN22* SNPs and disease risk. Genetic associations were examined in European-Americans (EA), African-Americans (AA), Asians (AS), and Hispanics (HS). Our results confirm SLE association with missense rs2476601 in EA, and we identified association of rs3765598 with SLE in HS. No significant associations were observed in AA or AS.

## Materials and Methods

### Ethics statement

Samples were supplied from multiple investigators from different institutions with approval from their respective institutional review boards (IRBs). All study participants provided written consent prior to study enrolment; consent forms were obtained at each location under IRB guidelines. Samples were then assembled at the Oklahoma Medical Research Foundation (OMRF) and the study protocols (including the enrollment process, consent forms, collection of DNA and subject information) for this study were approved by the Oklahoma Medical Research Foundation (OMRF) Institutional Review Board.

### Recruitment and Biological Sample Collection

We participated in the “*Large Lupus Association Study #2*” (LLAS2) in which >16,500 individuals were genotyped, including 8068 EA, 3462 AA, 2509 HS, and 2672 AS. Details of genotyping procedures have been described elsewhere [34]. All SLE cases met 1997 ACR classification criteria for SLE [35], [36]. Individual ethnicities were self-reported and genetic outliers were removed by principal component analysis (described below).

### Genotyping

All genotype data were generated using Illumina iSelect at the Oklahoma Medical Research Foundation (OMRF) genotyping facility. For this project, ten PTPN22 SNPs were generated using Illumina Genome Studio (http://www.illumina.com/software/genomestudio_software.ilmn). Following genotype scoring, SNP clusters were evaluated electronically using the Illumina BEADSTUDIO® software package (http://www.illumina.com). Ambiguous SNP clusters were evaluated manually and SNPs with poor cluster characteristics were not analyzed. All samples had 350 AIMs (ancestry informative markers) and >6000 “null” SNPs (not selected based on *P*-values, or known associated with any autoimmune disease, http://www.gwascentral.org [37]) genotyped to facilitate adjustment for genetic ancestry and population sub-structure.

### Quality Control

Quality control (QC) of the genotype data was performed before imputation. Subjects were excluded from analysis if they had <90% genotyping success or were population stratification outliers. Samples were evaluated for relatedness using identity by descent (IBD), and duplicate or related samples (IBD >0.25) were removed. Using AIMs and “null” SNPs, we performed principal component analysis (PCA, EIGENSTRAT [38], [39], and STRUCTURE [40] to identify outliers, hidden population structure, and estimate individual ancestry proportions as previously described [41]. SNPs were removed if they had (a) >10% missing genotypes, (b) showed evidence of deviation from Hardy-Weinberg equilibrium (P_HWE_<0.001 in controls or <0.0001 in cases), (c) for poor clustering, or had a minor allele frequency (MAF) <1%.

### Imputation-based Association Analysis

Imputation is a statistical method used to determine probabilistically missing or untyped genotypes using a densely mapped reference panel (26–28). We performed imputation-based analysis in our ethnic-specific case-control samples using publicly available 1000Genomes as data reference panels (2010–11 1000G Interim Phase I) for imputation, including 246 AA (YRI+LWK+ASW), 381 EA (GBR+FIN+IBS+CEU+TSI), 181 HS (PUR+CLM+MXL), and 286 AS (JPT+CHB+CHS). For HapMap family data only founders were included in the reference panels. From this region (114,356,663–114,418,205 bp) 527 SNPs were available. Imputation was performed using MACH [42], which provided a quantitative assessment of estimate uncertainty (Rsq). Allelic association results were adjusting for imputation uncertainty with mach2dat. All imputed SNPs were filtered based on QC measures (HWE >0.001, MAF >0.01, Rsq >0.6).

### Statistical Analyses for Assessing Genetic Association

At each SNP allelic association was assessed between cases and control using the χ^2^ test with 1 d.f. Allelic odds ratio (OR) and 95% confidence intervals (95% CIs) were calculated using PLINK [43]. Haploview [44] was used to estimate the linkage disequilibrium (LD) between markers in each ethnic population. We performed pair-wise conditional haplotype analysis using WHAP [45] to identify which associations were independent, and not due to LD with primarily associated SNPs. For each SNP we assessed whether it was still associated with SLE after conditioning on the most significant SNP. To correct for multiple testing, false discovery rate (FDR) methods were used and q values were calculated using PLINK (43). Q values correspond to the proportion of false positives among the results. Thus, q values <0.05 signify less than a 5% false positive rate and are taken as a measure of significance.

### ACR Classification Criteria and Sub-criteria Used for Association Analysis

Clinical data for the ACR classification criteria [36] for each participant was obtained through medical record review. ANA, anti-dsDNA, anti-Sm, anti-cardiolipin were determined in the CAP-certified OMRF Clinical Immunology Laboratory by an immunofluorescence (ANA, anti-dsDNA), immunodiffusion (anti-SM), or enzyme linked immunosorbent assay (aCL) at the enrollment time point [46]. Individual ACR criterion, or sub-criterion, including autoantibody status, were coded as positive, negative, or missing. SLE patients were grouped by the presence or absence of individual ACR criterion. Logistic regression and Pearson χ^2^ tests were used to assess statistical significance with associated SNPs in both case-only (all cases positive for given criteria versus all cases negative for the same criterion) and case-control (all cases positive for given criterion versus all healthy controls) analyses.

## Results

A total of 7427 EA (3936 cases and 3491 controls), 3338 AA (1527 cases and 1811 controls), 2525 AS (1265 cases and 1260 controls), and 2299 HS (1492 cases and 807 controls) passed QC and were used for association analyses. All ten genotyped SNPs passed QC in EA and HS, though rs2476601 was monomorphic in AS and AA, and rs33996649 failed genotyping in AA and AS. Using our genotypes and 1000Genomes Project data, 109 to 165 SNPs were imputed (165 in AA, 113 in EA, 109 in HS, and 101 in AS). Of these, a total of 107 SNPs passed QC (81 in AA, 83 in EA, 83 HS, and 79 AS).

**Figure 1:**
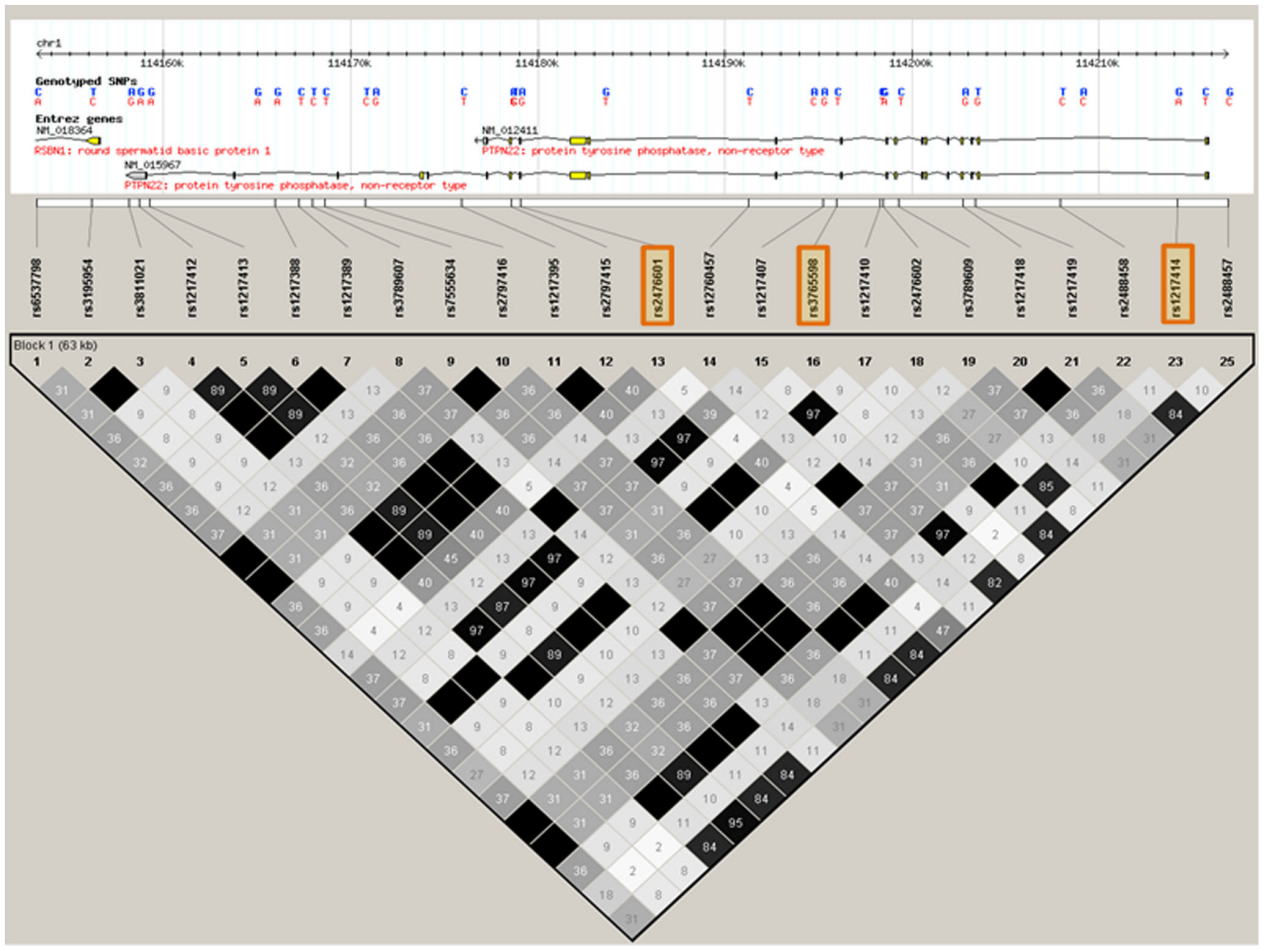
The haplotype structure of select SNPs from the *PTPN22* region in EA controls. Blocks connecting SNP pairs are shaded according to the strength of the linkage disequilibrium (r^2^).

Significant allelic association between *PTPN22* was observed in EA and HS (**Table 1**). In EA, 46 SNPs were associated (p<0.05) with SLE. The most significant association was with rs2476601 (P = 4.13×10^−9^, OR = 1.40 95% CI = 1.25–1.56) in EA. Intronic SNP rs1217414 was also significant in EA (P = 7.39×10^−4^, OR = 0.88 95% CI 0.82–0.95). **Figure 1** shows the overall haplotype structure from select SNPs in this genomic region in EA including the two isoforms of *PTPN22*. All imputed SNPs from the *PTPN22* region are shown in **Figure S1**. SNP rs2476601 was not significantly associated with SLE in HS (P = 0.20) and was monomorphic in AA and AS.

**Table 1:** Distribution of the minor allele frequency, case control association, and imputation-based association results for significantly associated SNPs in European-Americans, Hispanic, African-Americans, and Asians.

				EA (3936/3491)				HS (1492/807)				AA (1527/1811)				AS (1265/1260)			
SNP	BP	A	typed	F_A	F_U	P-val.	OR (95% CI)	F_A	F_U	P-val.	OR (95% CI)	F_A	F_U	P-val.	OR (95% CI)	F_A	F_U	P-val.	OR (95% CI)
rs2476601	114179091	A/G	genotyped	0.109	0.080	4.13E-9	1.40 (1.25–1.56)	0.054	0.045	0.1988	1.20 (0.91–1.60)	–	–	–	–	–	–	–	–
rs3765598	114214190	T/C	genotyped	0.188	0.186	0.7371	1.01 (0.93–1.10)	0.138	0.168	0.0066	0.79 (0.67–0.94)	0.089	0.085	0.5033	1.06 (0.89–1.26)	0.218	0.202	0.1683	1.10 (0.96–1.26)
rs1217414	114195986	A/C	genotyped	0.256	0.280	7.39E-4	0.88 (0.82–0.95)	0.180	0.191	0.3637	0.93 (0.80–1.09)	0.669	0.676	0.5460	0.97 (0.88–1.08)	0.071	0.081	0.1710	0.86 (0.70–1.07)

A1 = minor allele; F_A = minor allele frequency for SLE cases; F_U = minor allele frequency for controls.

In HS, 17 SNPs were significantly associated with SLE, all of which were of similar magnitude (P-values between 0.003–0.007, ORs between 0.79–0.82). Of these SNPs, rs3765598 (P = 0.007, OR = 0.79 95% CI = 0.67–0.94) was predicted to be an eQTL [47], which most likely affects binding and expression of PTPN22 in monocyte cell line [47].

To identify independent associations within significant signals in EA, we performed conditional haplotype analysis using WHAP [45]. This analysis showed that controlling for rs2476601 accounted for most of the association between *PTPN22* and SLE in EA (**Table S1**). However, since rs1217414 remained significant after being conditioned on rs2476601 (P = 0.022), and rs2476601 remained significant (P = 7.0×10^−7^) after conditioned on rs1217414, we performed haplotype analysis using these SNPs. The overall haplotype was significant (P_hap_ = 2.40×10^−9^) at a similar magnitude as individual SNP rs2476601 (P = 4.13×10^−9^). In fact, these SNPs are physically 35.6 kb apart and have a very low LD (r^2^ = 0.04), implying independence. The risk haplotype at both SNPs (AG) was the most significant (P = 9.58×10^−9^, OR = 1.38), and the non-risk haplotype (GA) was significant as well (P = 7.39×10^−4^, OR = 0.88). The haplotype which was non-risk for rs2476601/risk for rs1217414 was not significant (GG) (P = 0.63). When we compared the AG (both risk alleles) vs. GA (both non-risk alleles), the OR increased to 1.47 (p = 9.5×10^−9^). However, we did not observe the (AA) haplotype in our data.

To assess whether *PTPN22* genetic associations were enriched in SLE patients with specific clinical manifestations, a case-only analysis of the 11 ACR classification criteria and 5 autoantibodies was performed. Case-only analysis demonstrated association between rs2476601 and anticardiolipin IgG (aCL IgG >20) antibody using 121 patients positive compared to 578 SLE patients known to be negative for aCL IgG (P = 0.012, OR = 1.65 (95%CI = 1.11–2.46)) (**Table 2**). When adjusting for false discovery rate (FDR) this association remained significant, though the association was modest (FDR = 0.049). The allelic effect (OR) increased when these EA cases were compared to 3491 EA controls (P = 8.43×10^−5^, OR = 2.07, 95%CI = 1.42–2.99; FDR = 0.0008). The effect was further increased using 56 EA patients with high titer aCL IgG>40 (P = 9.67×10^−6^, OR = 2.79 95%CI 1.73–4.48; FDR = 9.67×10^−5^). The MAF of the ‘A’ allele increased from 8% in controls to 11% in all SLE cases, 15% in cases positive for aCL IgG, and 20% in cases with aCL IgG>40. Additional analyses suggest a positive correlation between risk allele ‘A’ and higher aCL IgG titer in 1251 available European samples (cases or controls) when aCL IgG titer used as a phenotype in quantitative analyses tests (qtl) (P = 0.009). Moreover, 42% of these aCL positive lupus patients are also diagnosed with anti-phospholipid syndrome (APS) with sufficient evidence of thrombosis (peripheral, cerebral, cardiac or pulmonary). We observed similar results in our EA population; 50 lupus patients with aCL-positive APS compared to healthy control (P = 0.003, OR = 2.17, 95%CI = 1.26–3.74; FDR = 0.03). A similar trend was observed in Hispanics but the number of samples was not sufficient (data not shown). However, there was no association between rs2476601 and the presence of lupus anticoagulant (LA) with negative or low titer aCL (P = 0.09). While not statistically significant, we observed an increase in the odds ratio in Europeans for the presence of lupus nephritis, or anti-dsDNA antibodies (1.49 and 1.47 respectively) for rs2476601.

**Table 2:** Clinical criteria sub-analysis results for European-Americans and Hispanics.

											case-only			case positive-control		
Population	Criteria	SNP	A1	A2	# pos	#neg	# ctrl	F_P	F_N	F_U	P-value	FDR	OR (95% CI)	P-value	FDR	OR (95% CI)
EA	aCL IgG	rs2476601	A	G	121	578	3491	0.156	0.100	0.080	0.0120	0.0492	1.65 (1.11–2.46)	2.69E-5	2.23E-3	2.11 (1.48–3.02)
HS	anti-nRNP	rs2476601	A	G	108	229	807	0.083	0.044	0.045	0.0372	0.8763	1.99 (1.03–3.85)	0.0019	0.9671	1.90 (1.07–3.37)
HS	malar rash	rs3765598	T	C	316	169	807	0.152	0.086	0.168	0.0034	0.6823	1.91 (1.23–2.96)	0.6136	0.0770	0.95 (0.73–1.25)

F_P = frequency for SLE cases positive for sub-criteria, F_N = frequency for SLE cases negative for sub-criteria, F_U = frequency for controls.

We also found that EA lupus patients with co-existent thyroid disease strengthened the observed association result. The OR for SNP rs2476601 was increased to OR = 2.07 (95%CI 1.44–2.99), when 117 European cases with co-existent thyroid disease were compared with 3491 European controls with a MAF of 15% in cases in compare to 8% in controls (P = 6.38×10^−5^; FDR = 0.001). Among these, only 24 (20%) had aCL IgG>20 and in a separate analysis, lupus patients with co-existent thyroid disease and aCL IgG<20 (93 patients) still shows similar increased risk (P = 0.001, OR = 1.94 95%CI 1.27–2.95). In case-only analyses when these cases were compared with 514 SLE patients with no history of thyroid disease a similar trend was obtained (P = 0.02, OR = 1.60, 95%CI = 1.06–2.39).

Although rs2476601 was not significantly associated with SLE (P = 0.20) in HS, this SNP was marginally significant with anti-nRNP positive SLE cases (P = 0.037, OR = 1.99; 108 cases positive, 229 cases negative) in case-only analysis (**Table 2**). For anti-nRNP positive cases versus healthy controls association remained significant (P = 0.002, OR = 1.90). MAF of the ‘A’ allele increased from 4.6% in controls to 8.3% in anti-nRNP positive cases. SNP rs3765598 was associated with malar rash (P = 0.003, OR = 1.91; 316 cases positive, 169 cases negative) but this result was not confirmed in case positive vs. controls (P = 0.61, OR = 0.95). While neither of the case-only results were significant after adjusting for FDR (P_nRNP-rs2476601_ = 0.88, P_malar-rs3765598_ = 0.68), the sample sizes for these analyses were quite small and lacked statistical power. Notable, SLE cases negative for malar rash had the lowest MAF (MAF_CONTROL_ = 16.8%, MAF_NEG_ = 8.6%, MAF_POS_ = 15.2).

**Figure 2:**
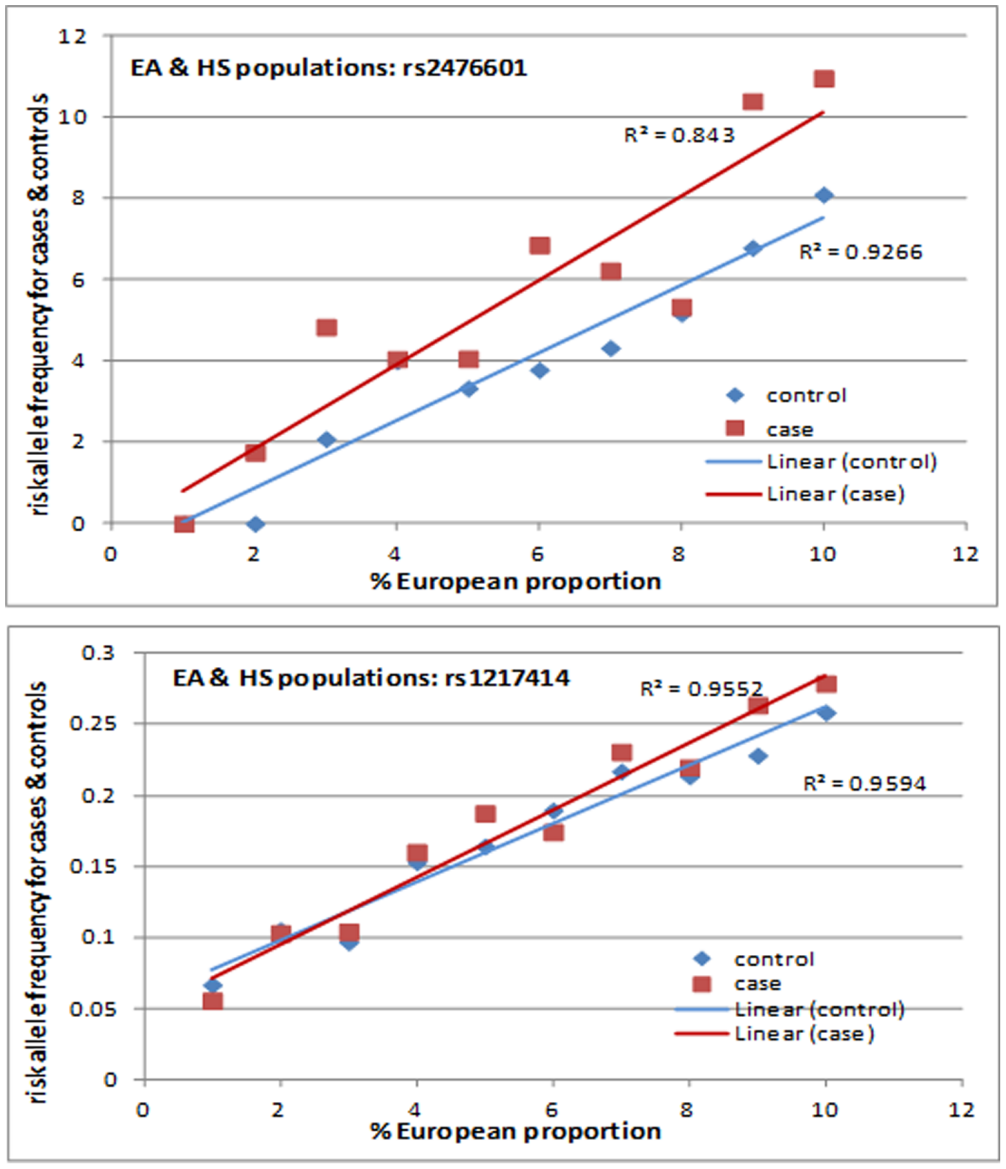
Minor allele frequency of rs2476601 and rs1217414 as a function of proportion of European admixture for SLE cases and healthy controls.

We assessed (a) whether MAF is correlated with individual European admixture, and (b) the impact of European admixture in association. We only included EA and HS for this analysis since these populations were polymorphic for this SNP. Indeed, MAF increased for the rs2476601 risk allele (A) as the proportion of European Admixture increased (**Figure 2**). Next, individuals were grouped by European admixture proportion and allelic association was performed for each group. For individuals with low proportions of European admixture (below 40%; 157 cases and 132 controls) and those with moderate proportions of European admixture (40–90%; 662 cases and 380 controls) rs2476601 was not associated with SLE, P_low_ = 0.60 and P_moderate_ = 0.10 respectively. For individuals with the highest proportion of European admixture (>90%; 3648 cases and 3450 controls) this SNP was significantly associated with SLE (P_high_ = 4.84×10^−9^). Similar results were found for rs1217414 (P_low_ = 0.40, P_moderate_ = 0.78, P_high_ = 0.007).

We also performed this analysis based on proportion of Native American ancestry. Individuals with low (<10%; 3870 cases and 3572 controls) or moderate proportion (10–30%; 255 cases and 131 controls) of Native American ancestry did not show SLE association with rs3765598, P_low_ = 0.70 and P_moderate_ = 0.99 respectively. However, individuals with relatively higher proportions of Native American ancestry (>30%; 342 cases and 259 controls) did show significant association with SLE (P_high_ = 0.018), despite the relatively small sample size.

## Discussion

We genotyped ten SNPs in *PTPN22*, including two missense (rs2476601 (R620W), rs33996649 (R263Q)) and 5′-UTR (rs2488457) SNPs, in four large multi-ethnic populations. Using these genotypes and HapMap data we imputed 107 SNPs in diverse ethnic groups to elucidate the effect size and potential relation of these variants with SLE risk across populations. Our results were consistent with previous reports: we reinforced that SLE-association with *PTPN22* is largely accounted for by rs2476601 in individuals of European ancestry [1], [48], [49], [50]. While there is a strong North-South gradient of the risk allele frequency across Europe [26], we did not have sufficient AIMs determine North vs. South European ancestry we could not assess the effect of this gradient in our study. We were unable to detect association with rs2476601 in AA or AS, or any other SNP within *PTPN22*. This result is consistent with earlier reports that this SNP is rare and not associated with SLE in AA [3], [51] or in Han Chinese [52].

Recent reports continue to shed light on mechanisms of non-synonymous rs2476601 (R620W). *PTPN22* encodes the protein tyrosine phosphotase which has an important regulatory role in multiple signaling pathways and functions to dephosphorylate specific tyrosine residues in target proteins. The R620W mutation changes the highly conserved amino acid arginine to tryptophan which disrupts the P1 proline-rich motif and could cause accelerated degradation of the tyrosine phosphotase protein [53], [54]. TCR signaling has been shown to be reduced in T cells containing the disease-susceptibility allele [17]. Protein degradation may lead to hyper-responsiveness of lymphocyte and dendritic cells, which could help explain increased risk for autoimmunity [54]. Additionally, the *PTPN22* risk genotype (R620W) may also alter B cell signaling transduction either through decreases in B cell proliferation or a deficit in phosphorylation of key signaling proteins [20]. Recent murine studies provide important new insight regarding the role of *PTPN22* (R620W) risk allele in regulation of signal transduction and maintenance of immune tolerance. Interestingly, when the analog mutation of (R620W) has been knocked into the murine ortholog of the gene, this mutation did not influence the half-life of PTPN22 protein as previously suggested [54] but altered receptor-mediated protein tyrosine phosphorylation and caused hypersensitivity to antigen receptor stimulation. Indeed, B cell expression of the risk variant was sufficient to promote systemic autoimmunity [55], [56].

After controlling for rs2476601 using conditional haplotype analysis we found that it accounted for most of the association in *PTPN22*. However, even after conditioning on rs2476601 there was a modest association of rs1217414 with SLE. rs1217414 has been implicated in early onset psoriasis [57] and RA [58], [59] and may be involved in autoimmunity predisposition. Additionally, bioinfomatic search found that this SNP is potentially in an active promoter site for a chromatin regulatory site for B-lymphocyte cells [54].

While non-synonymous rs33996649 (R263Q) did not pass the threshold for significance, the P-value was suggestive (P = 0.08) in EA. This SNP, which lies within the catalytic domain of enzyme, is correlated with *PTPN22* gene expression [60], leads to reduced phosphatase activity [61] and was negatively associated with SLE in Europeans [61]. An association independent of rs2476601 was identified for rs1217414 and rheumatoid arthritis in populations of European and Asian descent. Our data does not support a significant association of this SNP with SLE in non-European derived populations (**Table S1**).

In HS we observed association of potential eQTL rs3765598 with SLE. This SNP is in a potential enhancer of regulatory chromatin site for CD4+CD25−CD45RO+ memory primary cells. Although the minor allele for rs3765598 is less common in Yorubans (MAF = 4%), and is more common in individuals of European descent (CEU MAF = 23%), it was not associated in our EA population (P = 0.75).

We confirmed associations between *PTPN22* SNPs with selected autoantibody sub-phenotypes and with malar rash in independently associated SNPs. We avoided inter-laboratory variation by only considering data from OMRF testing laboratory. When analyzing subgroups of SLE cases based upon clinical criteria, there is usually a loss of power due to the smaller sample size relative to all SLE. Alternatively, we can increase statistical power by analyzing more homogenous groups when clinical phenotype is more strongly associated compared to SLE. Because of these issues using P-values alone is unreliable; therefore we look for an increasing magnitude of the OR. In this report EA patients positive for IgG anti-cardiolipin antibodies (aCL IgG) were enriched for the risk allele (A) and effect sizes increased (OR = 1.30 for SLE cases vs. controls, OR = 1.62 for case-only). This was especially true for those with moderate to high anticardiolipin antibodies (aCL IgG>40) as well as a subset of APS patients who also were aCL positive (see above). This effect was not detected in patients with low titer aCL or presence of only lupus anticoagulant. ACL antibodies can be found in patients with a wide spectrum of clinical conditions however the higher titers of these antibodies are more commonly seen in APS. To our knowledge the potential association of this SNP among patients with primary or secondary APS has not been reported previously. A recent study in a Polish population with endometriosis, only those with positive anti-cardiolipin antibodies and anti-nuclear antibodies were associated with rs2476601 (P = 0.009). However, the number of samples was small and it is not clear whether any of these patients suffered from co-existent APS [62]. It has been reported in multiple lupus studies [63], [64] that the presence of aCL (IgG) antibodies in lupus patients especially those with APS syndrome, are associated with poor prognosis, increased mortality, and an earlier time of onset of several criteria, in particular malar rash and nephritis. This could be related to thrombotic events in kidney, vasculitis, arterial occlusions and coexistent thrombocytopenia. Given the notable increase of OR between general SLE (OR_SLE_ = 1.40) and SLE patients with APS (OR = 2.17), rs2476601 could be a surrogate of lupus severity at least in European ancestry. It is noteworthy that although availability of data for specific autoantibodies, in particular aCL, were limited in our cohorts, there was no evidence of heterogeneity or inconsistency in risk ratio estimates between cohorts or any evidence of site-specific confounding effect. Further studies are necessary to confirm or refute this possibility.

In summary, in accordance with previous studies, our data on multiethnic populations further confirmed that rs2476601 (R620W) is unequivocally associated with SLE in individuals with European ancestry. Additionally, since rs2476601 was associated with aCL IgG our data suggests that this *PTPN22* variant might be associated with pathogenesis of antiphospholipid syndrome in Europeans. We also detected significant association with rs3765598 in HS, though no significant associations were observed in AA or AS. Further, independent replication studies are required in these populations to establish these novel associations with SLE and its clinical sub-phenotypes and autoantibody status.

## Supporting Information

Figure S1 The haplotype structure of all imputed SNPs in the *PTPN22* region in EA controls. Blocks connecting SNP pairs are shaded according to the strength of the linkage disequilibrium (r^2^).
(DOCX)

Table S1 Distribution of the minor allele frequency, case-control association, and imputation-based association results for all SNPs for European-Americans, Hispanics, African-Americans, and Asians.
(XLSX)
