# Supplementary material for: PTPN22 Association in Systemic Lupus Erythematosus (SLE) with Respect to Individual Ancestry and Clinical Sub-Phenotypes
Source: PLoS One. 2013 Aug 7;8(8):e69404. doi: 10.1371/journal.pone.0069404 (PMC3737240; doi:10.1371/journal.pone.0069404)
Supplement: Supplementary file 1 [file pone.0069404.s001.docx]

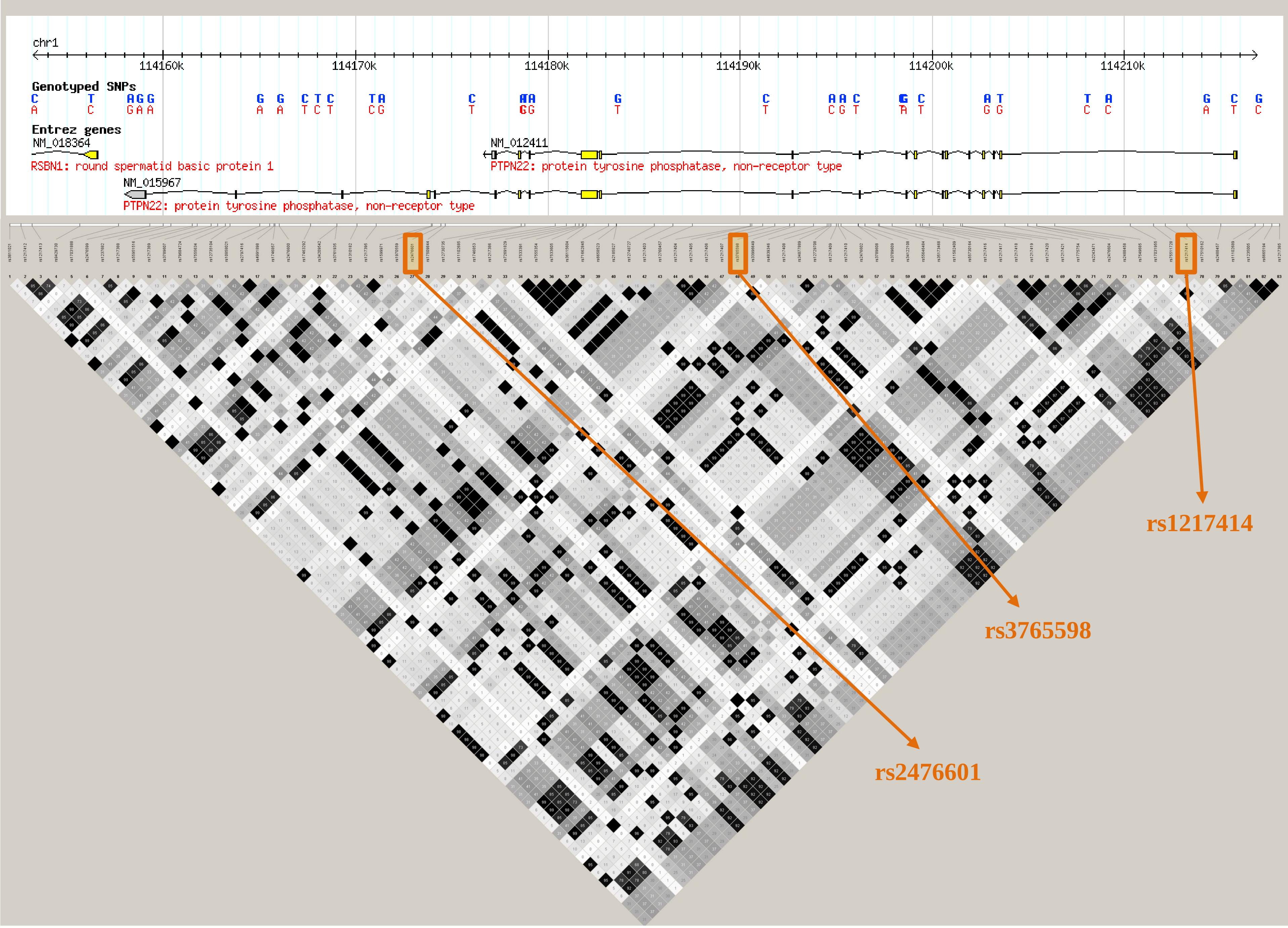
chr1
114160k
114170k
114180k
114190k
114200k
114210k
Genotyped SNPs
Entrez genes
NM_018364
RSBN1: round spermatid basic protein 1
NM_012411
PTPN22: protein tyrosine phosphatase, non-receptor type
NM_015967
PTPN22: protein tyrosine phosphatase, non-receptor type
rs1217414
rs3765598
rs2476601
